# Antioxidant, Anti-inflammatory, and Antiulcer Potential of Manuka Honey against Gastric Ulcer in Rats

**DOI:** 10.1155/2016/3643824

**Published:** 2015-12-07

**Authors:** Saad B. Almasaudi, Nagla A. El-Shitany, Aymn T. Abbas, Umama A. Abdel-dayem, Soad S. Ali, Soad K. Al Jaouni, Steve Harakeh

**Affiliations:** ^1^Biology Department, Faculty of Science, King Abdulaziz University, Jeddah 21589, Saudi Arabia; ^2^Department of Pharmacology and Toxicology, Faculty of Pharmacy, King Abdulaziz University, Jeddah 21589, Saudi Arabia; ^3^Department of Pharmacology and Toxicology, Faculty of Pharmacy, Tanta University, Tanta 31111, Egypt; ^4^Special Infectious Agents Unit, King Fahd Medical Research Center, King Abdulaziz University, Jeddah 21589, Saudi Arabia; ^5^Biotechnology Research Laboratories, Gastroenterology Surgery Center, Mansoura University, Mansoura 35516, Egypt; ^6^Animal Facility Unit, King Fahd Medical Research Center, King Abdulaziz University, Jeddah 21589, Saudi Arabia; ^7^Anatomy Department (Cytology and Histology), Faculty of Medicine, King Abdulaziz University, Jeddah 21589, Saudi Arabia; ^8^Department of Hematology and Yousef Abdulatif Jameel Chair of Prophetic Medicine Application, Faculty of Medicine, King Abdulaziz University, Jeddah 21589, Saudi Arabia

## Abstract

Gastric ulcers are among the most common diseases affecting humans. This study aimed at investigating the gastroprotective effects of manuka honey against ethanol-induced gastric ulcers in rats. The mechanism by which honey exerts its antiulcer potential was elucidated. Four groups of rats were used: control, ethanol (ulcer), omeprazole, and manuka honey. Stomachs were examined macroscopically for hemorrhagic lesions in the glandular mucosa, histopathological changes, and glycoprotein detection. The effects of oxidative stress were investigated using the following indicators: gastric mucosal nitric oxide (NO), reduced glutathione (GSH), lipid peroxide (MDA, measured as malondialdehyde) glutathione peroxidase (GPx), superoxide dismutase (SOD), and catalase. Plasma tumour necrosis factor-*α*, interleukin-1*β*, and IL-6 were also measured. Manuka honey significantly decreased the ulcer index, completely protected the mucosa from lesions, and preserved gastric mucosal glycoprotein. It significantly increased gastric mucosal levels of NO, GSH, GPx, and SOD. Manuka honey also decreased gastric mucosal MDA and plasma TNF-*α*, IL-1*β*, and IL-6 concentrations. In conclusion, manuka honey likely exerted its antiulcer, effect by keeping enzymatic (GPx and SOD) and nonenzymatic (GSH and NO) antioxidants as well as inflammatory cytokines (TNF-*α*, IL-1*β*, and IL-6) in a reduced form, inhibited lipid peroxidation (MDA), and preserved mucous glycoproteins levels.

## 1. Introduction

Gastric ulcers have long been rated as one of the most common diseases affecting humans in general and young people in particular [1]. There are several drug categories that have been used in the treatment of gastric ulcers, including proton pump inhibitors, M1-receptor blockers, and H2-receptor antagonists [2]. There are numerous side effects associated with the drugs used in the treatment of ulcers, including arrhythmia, impotence, gynaecomastia, and hematopoietic changes. Moreover, there is a very high relapse rate (80% at 1st year and 100% in the 2nd year of treatment). Other issues include the long-term duration of the treatment period (therapy with H2-receptor antagonists for 1 year) and the incomplete eradication of ulcers. Therefore, new treatments have been sought to enhance the efficacy of current drugs or to discover potential new agents that are more effective and less expensive and have fewer health-associated side effects than those currently used [3].

Ethanol is a well-known damaging agent to the gastric mucosa, used in animals and clinical studies [4]. The model of an ethanol-induced gastric ulcer is used for the evaluation of the gastroprotective activity of many new therapeutics and natural products [5]. Ethanol results in a rush in neutrophil infiltration into the site of injury, which is essentially an acute inflammatory reaction. This is followed by a surge in the formation of reactive oxygen species (ROS), which cause oxidative bursts in the essential cellular components, including nucleic acids, lipids, and proteins [6]. Ethanol also induces alterations in the cytokine balance responsible for inflammation in the gastric mucosa [7]. The proinflammatory cytokine tumour necrosis factor-*α* (TNF-*α*) was found to play an important role in ethanol-induced apoptosis during gastric ulcer formation [8].

Manuka honey is rich with flavonoids. Flavonoids-polyphenolic compounds are a group of secondary metabolites naturally occurring in the plant kingdom, possess numerous pharmacological activities (antiinflammatory, antimicrobial and gastroprotective), and prevent gastric ulcer formation through several mechanisms, including antisecretory and antioxidant mechanisms [9].

Manuka honey is a unifloral honey derived from the manuka tree,* Leptospermum scoparium*, belonging to the family Myrtaceae in New Zealand and the Eastern region of Australia [10]. It is a dark honey and has attracted a lot of attention, especially in regard to its antimicrobial agent, antioxidant efficacy, and potential role in wound healing [11, 12]. Compared to other honey types, manuka honey contains the highest amount of phenolic and flavonoid compounds (pinobanksin, pinocembrin, and chrysin) that have been identified with potent ROS scavenging activity [13–15].

This study aimed at investigating the gastroprotective effects of manuka honey against ethanol-induced gastric ulcers in rats using omeprazole as a control drug. In addition, the mechanism by which honey exerts its efficacy is elucidated in terms of oxidative stress measures and inflammatory cytokine production response.

## 2. Materials and Methods

### 2.1. Animals

Twenty-four, 6-week-old male albino rats weighing between 220 and 250 g were used in this study. The animals were housed in the animal facility at King Fahd Medical Research Center, King Abdulaziz University, Jeddah, Saudi Arabia, under a 12 h light/dark cycle at a temperature of 25°C and relative humidity ranging from 60 to 70% throughout the experiment. The animals had free access to diet and water ad* libitum*.

Prior to the induction of gastric ulcer, animals were fasted for 36 h to ensure an empty stomach (water was allowed). The animals were individually housed in wire mesh cages to avoid coprophagy. The use of experimental animals was conducted in strict compliance with the rules and regulations established by the Research Ethics Committee at King Abdulaziz University after obtaining their ethical approval to pursue this study.

### 2.2. Honey and Omeprazole

Royal Bee 20 + active manuka honey 100% (Royal Bee, New Zealand) was used in this study. Omeprazole was obtained from Sigma, USA. Powdered omeprazole and liquid honey were individually reconstituted in a 3% v/v tween 80 to prepare a 10% stock solution [16]. Stock solutions were freshly prepared daily and used for feeding.

### 2.3. Induction of Ulcer

The induction of ulcer was achieved by oral administration (p.o.) of absolute ethanol at a dose of 1 mL/200 g body weight by intragastric gavage, as described elsewhere [17]. The rats were killed, 1 h later by cervical dislocation after being anesthetized [18].

### 2.4. Treatment Groups

Animals were randomly divided into 4 groups (6 rats in each):

1. Control: rats in this group received tween 80, p.o. (3%).
2. Ethanol (ulcer): rats in this group received ethanol (1 mL/200 g body weight, p.o.) [16].
3. Omeprazole: rats in this group received omeprazole 7 days before induction of ulcer at a dose of 40 mg/kg, p.o. [19, 20].
4. Manuka honey: rats in this group received manuka honey 7 days before induction of ulcer at a dose of 0.1, 1.0, and 2.5 gm/kg, p.o. [21, 22].

### 2.5. Measurement of Body Weight Gain (BWG) and Food and Water Consumption

The body weight gain, total food pellet consumption, and water intake of the rats in both the control and the manuka honey (2.5 gm/kg) groups were recorded daily during the experiment.

### 2.6. Gross Examination of Gastric Mucosa

After sacrificing the animals, the stomachs were removed and washed with 0.9% saline solution to clean away the blood. This was followed by macroscopic examination of the stomach for the detection of any hemorrhagic lesions on the glandular mucosa. The length in mm of each lesion was measured to determine the mean ulcer index (UI) [22]. The severity of mucosal lesions was scored as follows: no ulcer (0), small ulcer (1-2 mm) (1), medium ulcer (3-4 mm) (2), large ulcer (5-6 mm) (4), and huge ulcer (>6 mm) (8). The UI was determined by adding the sum of the total of the scores and dividing by the number of animals [23].

### 2.7. Histopathological Examination of Gastric Mucosa

Each freshly excised clean stomach was divided into two parts. One of the parts was used for the histopathological examination and glycoprotein determination. The other part was stored at −80°C pending biochemical analyses. For histopathological examination, tissues were fixed in a 10% buffered formalin solution. Formalin-fixed stomach sections were embedded in paraffin wax and serially sectioned (3–5 *μ*m) for further examination. One part was stained with hematoxylin and eosin (H&E) and observed for pathological changes using ordinary light microscopy.

### 2.8. Mucosal Glycoprotein Detection

For mucosal glycoprotein detection, paraffin sections were stained with periodic acid Schiff (PAS) for each rat in each group and examined using ordinary light microscopy.

### 2.9. Biochemical Analysis

A frozen portion of the stomachs was thawed and used for the determination of antioxidant levels. For SOD determination, thawed tissues were homogenized in 2% Triton X-100 containing a 0.32 M sucrose solution. Other stomach portions were homogenized in 50 Mm potassium phosphate, pH 7.5 and 1 Mm EDTA for MDA, GSH, NO, GPx, and CAT. Plasma samples were used for TNF-*α*, IL-1*β*, and IL-6 measurements. Serum was used for glucose, triglycerides (TG), total cholesterol, HDL-cholesterol, and LDL-cholesterol measurements. Homogenized tissues were twice subjected to a sanitation procedure, with 30 s intervals at 4°C. After the sonication process, homogenized tissues were centrifuged at 4000 rpm/min for 10 min at 4°C.

### 2.10. Measurement of Serum Glucose, Triglycerides (TG), Total Cholesterol, HDL-Cholesterol, and LDL-Cholesterol

Serum levels of glucose, triglycerides (TG), total cholesterol, HDL-cholesterol, and LDL-cholesterol were determined using an Auto Analyser (DIMENSION VISTA 1500, SIEMENS, Germany).

### 2.11. Measurement of Gastric Mucosal Nitric Oxide (NO)

NO was measured using Biodiagnostic kits (Egypt) according to Tarpey et al. [24]. Initially, nitrate was reduced to nitrite using the nitrate reductase enzyme. This was followed by an assay of the nitrite using Griess reagent at an optical density of 550 nm. Gastric mucosal NO concentration was expressed as *μ*mol/g tissue.

### 2.12. Measurement of Gastric Mucosal Reduced Glutathione (GSH)

GSH was quantified using Biodiagnostic kits (Egypt), which was based on the method developed by Ellman [25]. The gastric mucosal GSH concentration was expressed as U/g tissue.

### 2.13. Measurement of Gastric Mucosal Lipid Peroxide Measured as Malondialdehyde (MDA)

Gastric mucosal MDA was measured using Biodiagnostic kits (Egypt) according to Uchiyama and Mihara [26]. The color formed was measured at an optical density of 535 nm. The gastric mucosal MDA concentration was expressed as nmol/g tissue.

### 2.14. Measurement of Gastric Mucosal Glutathione Peroxidase Enzyme Activity (GPx)

Gastric mucosal GPx activity was measured using Biodiagnostic kits (Egypt). GPx activity was determined by measuring the rate of NADPH oxidation at 340 nm using H_2_O_2_ as the substrate [27]. GPx activity was expressed in U/g tissue.

### 2.15. Measurement of Gastric Mucosal Superoxide Dismutase Enzyme Activity (SOD)

Gastric mucosal SOD activity was measured using Biodiagnostic kits (Egypt) according to Nishikimi et al. [28]. This assay depends on the ability of the SOD to inhibit the phenazinemethosulphate-mediated reduction of nitrobluetetrazolium dye. SOD activity was expressed in U/mg tissue.

### 2.16. Measurement of Gastric Mucosal Catalase Enzyme Activity (CAT)

CAT activity was measured using Biodiagnostic kits (Egypt) according to Aebi [29]. H_2_O_2_ reacts with CAT. The test is based on the reaction of H_2_O_2_ with 3,5-dichloro-2-hydroxybenzene sulfonic acid and 4-aminophenazone, producing a colored chromophore that was measured at 510 nm. CAT activity was expressed in U/g tissue.

### 2.17. Measurement of Plasma Tumour Necrosis Factor-*α* (TNF-*α*), Interleukin-1*β* (IL-1*β*), and IL-6

TNF-*α*, IL-1*β*, and IL-6 levels were measured in an ELISA assay with Assaypro TNF-*α*, IL-1*β*, and IL-6 kits (30 Triad South Drive, St. Charles, MO 63304, USA) using monoclonal antibodies specific for TNF-*α*, IL-1*β*, and IL-6, respectively. Cytokine concentrations were calculated using standard purified recombinant cytokines.

### 2.18. Statistical Analysis

All data were presented as mean ± SD. Statistical software SPSS 20.0 was utilized. The results were statistically analyzed using a one-way analysis of variance (ANOVA) test. Statistical differences of *P* ≤ 0.05 were considered to be significant.

## 3. Results

### 3.1. Effect of Manuka Honey on % Body Weight Gain (BWG) and Food and Water Consumption

Treatment of rats with manuka honey (2.5 g/kg, p.o.) for 7 days caused a nonsignificant change in % BWG (*P* = 0.782), daily food consumption (*P* = 0.131), and water intake (*P* = 0.058) as compared to the control rats (Table 1).

### 3.2. Effect of Manuka Honey on Serum Glucose, Triglycerides (TG), Total Cholesterol, HDL-Cholesterol, and LDL-Cholesterol

Treatment of rats with manuka honey (2.5 g/kg, p.o.) for 7 days caused a nonsignificant change in serum glucose (*P* = 0.747), triglycerides (TG) (*P* = 0.686), total cholesterol (*P* = 0.460), HDL-cholesterol (*P* = 0.391), and LDL-cholesterol (*P* = 0.409) as compared to the control rats (Table 2).

### 3.3. Effect of Manuka Honey on the Severity of Gastric Lesion (UI)

**Figure 1:**
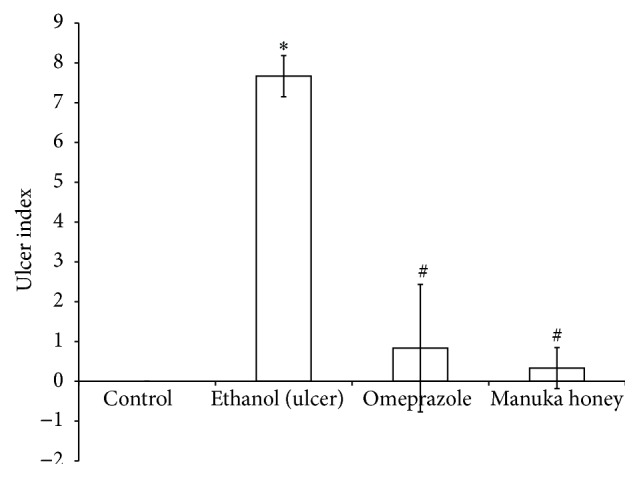
Effect of manuka honey on the severity of gastric lesion (ulcer index) measured in ethanol-induced gastric ulceration model. Ethanol treated rats were pretreated with either omeprazole (40 mg/kg) or manuka honey (2.5 g/kg). Each value is the mean ± SD (*n* = 6). ^*∗*^Significant versus control group (*P* ≤ 0.05). ^#^Significant versus ethanol (*P* ≤ 0.05).

**Figure 2:**
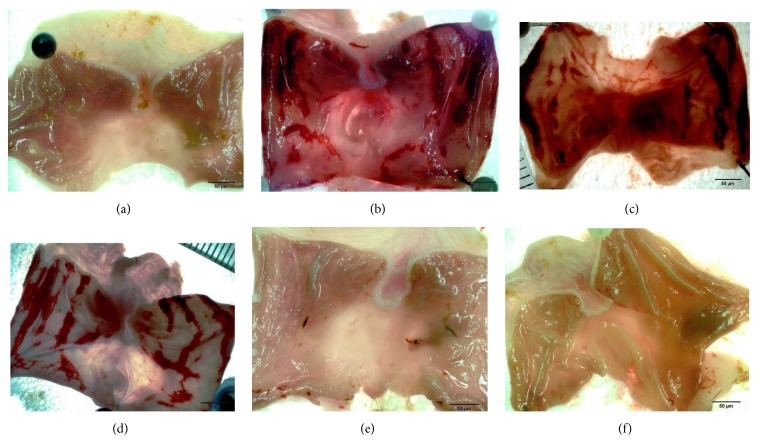
Effect of different doses of manuka honey on the severity of gastric lesion (gross examination) examined in ethanol-induced gastric ulceration model. (a) Control: intact gastric mucosa tissues; (b) ethanol (ulcer): severe lesions are seen with extensive visible haemorrhagic necrosis of gastric mucosa; (c) manuka honey (0.1 g/kg): severe lesions are seen with extensive visible haemorrhagic necrosis of gastric mucosa; (d) manuka honey (1.0 g/kg): severe lesions are seen with extensive visible haemorrhagic necrosis of gastric mucosa; (e) omeprazole: mild lesions of gastric mucosa are observed compared to the lesions in ethanol (ulcer); (f) manuka honey (2.5 g/kg): nearly normal gastric mucosa tissues. These photographs are typical of such tissues.

Treatment of rats with ethanol (1 mL/200 g, p.o.) caused a significant increase in the UI as compared to the controls (*P* = 0.000) (Figure 1). The pretreatment with either omeprazole (40 mg/kg, p.o.) or manuka honey (2.5 g/kg, p.o.) in ethanol-injected rats significantly decreased the UI by 89% and  96%, respectively, as compared to the ethanol-injected rats (*P* = 0.00) (Figure 1). However, pretreatment using lower concentrations of manuka honey (0.1 and 1.0 g/kg) resulted in no protection (Figures 2(c) and 2(d)).

### 3.4. Effect of Manuka Honey on the Severity of Gastric Lesion (Gross Examination) 

Treatment of rats with ethanol caused severe lesions with extensive visible hemorrhagic necrosis of gastric mucosa (Figure 2(b)). Pretreatment of ethanol-injected rats with omeprazole decreased the gastric mucosal lesions as compared to ethanol-induced lesions (Figure 2(e)). Pretreatment of ethanol-injected rats with manuka honey showed no protection. On the other hand, pretreatment of ethanol-injected rats with manuka honey (2.5 g/kg) provided significant protection of the mucosa from ethanol-induced lesions (Figure 2(f)).

### 3.5. Effect of Manuka Honey on the Gastric Mucosal Glycoprotein Formation Detected by PAS Staining

**Figure 3:**
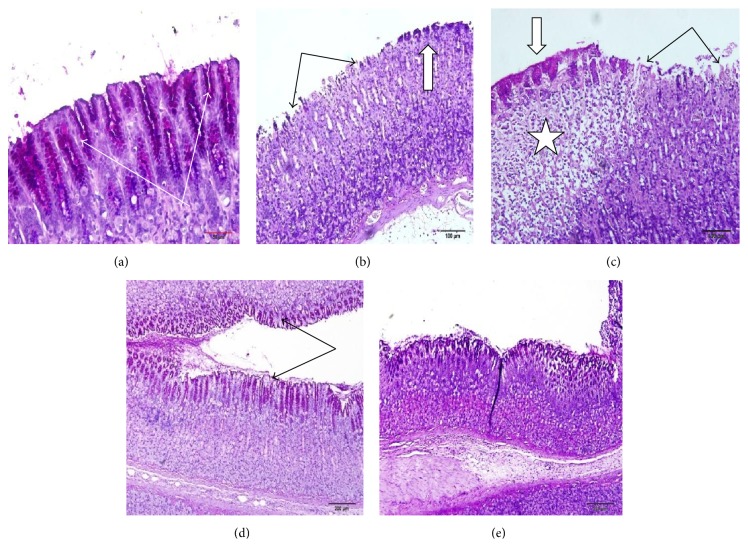
Effect of manuka honey on the gastric mucosal glycoprotein formation detected by PAS staining in the ethanol-induced gastric ulceration model. (a) Control; (b and c) ethanol (ulcer): marked glycoprotein depletion with a compensatory increase in nearby cells; (d) omeprazole: preserved gastric mucosal glycoproteins; (e) manuka honey: preserved gastric mucosal glycoproteins (sections are PAS stained ×20).

Treatments of rats with ethanol caused a marked depletion of gastric mucosal glycoprotein (Figure 3). Pretreatment with both omeprazole and manuka honey in ethanol-injected rats preserved gastric mucosal glycoproteins (Figure 3).

### 3.6. Effect of Manuka Honey on the Gastric Mucosal Histopathological Changes Detected by H&E Staining

**Figure 4:**
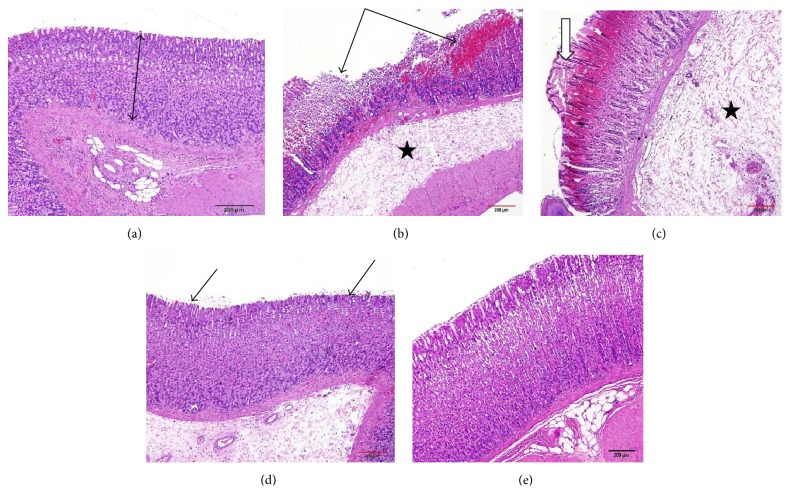
Effect of manuka honey on the gastric mucosal histopathological changes detected by H&E staining in ethanol-induced gastric ulceration model. (a) Control: intact mucosal layers; (b and c) ethanol (ulcer): ulcer with marked maceration of gastric mucosa; necrosis and hemorrhage (arrows). In some animals there is coagulative necrosis of superficial layers (white arrows) and evidence of submucosal widening indicating edema (stars); (d) omeprazole: normal gastric glands with focal loss of superficial mucous cells and hemorrhagic spots; (e) manuka honey: apparently normal mucosa (sections are H&E stained ×20).

Treatments of rats with ethanol caused ulcer formation with marked maceration of gastric mucosa, necrosis, and hemorrhage. In addition, in some animals, there was coagulative necrosis of superficial mucosal layers and evidence of submucosal widening, indicating edema (Figure 4). Pretreatment of ethanol-injected rats with omeprazole resulted in mild histopathological changes as compared to ethanol-treated rats. Normal gastric glands with a focal loss of superficial mucous cells and hemorrhagic spots were observed in omeprazole rats (Figure 4). On the other hand, pretreatment of ethanol-injected rats with manuka honey resulted in complete protection against ethanol-induced histopathological changes (Figure 4).

### 3.7. Effect of Manuka Honey on Gastric Mucosal Nitric Oxide (NO), Reduced Glutathione (GSH), and Lipid Peroxides (MDA) Concentrations

**Figure 5:**
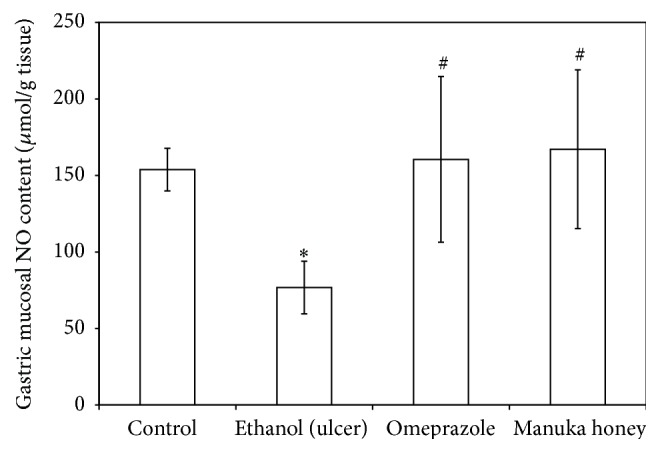
Effect of manuka honey on gastric mucosal NO content (*μ*mol/g tissue) measured in ethanol-induced gastric ulceration model. Ethanol treated rats were pretreated with either omeprazole (40 mg/kg) or manuka honey (2.5 g/kg). Each value is the mean ± SD (*n* = 6). ^*∗*^Significant versus control group (*P* ≤ 0.05). ^#^Significant versus ethanol (*P* ≤ 0.05).

**Figure 6:**
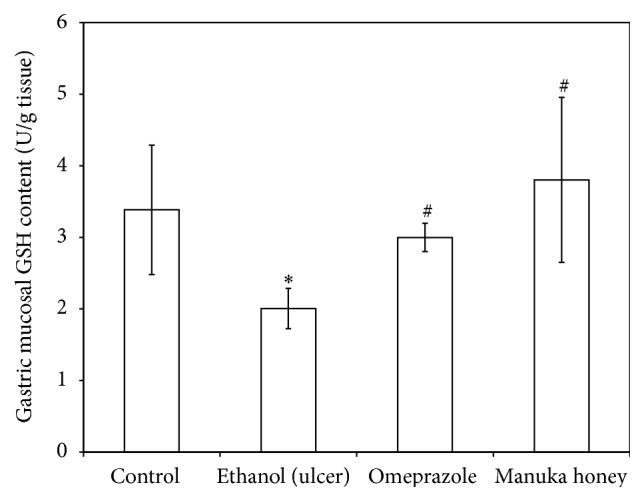
Effect of manuka honey on gastric mucosal GSH content (U/g tissue) measured in ethanol-induced gastric ulceration model. Ethanol-treated rats were pretreated with either omeprazole (40 mg/kg) or manuka honey (2.5 g/kg). Each value is the mean ± SD (*n* = 6). ^*∗*^Significant versus control group (*P* ≤ 0.05). ^#^Significant versus ethanol (*P* ≤ 0.05).

**Figure 7:**
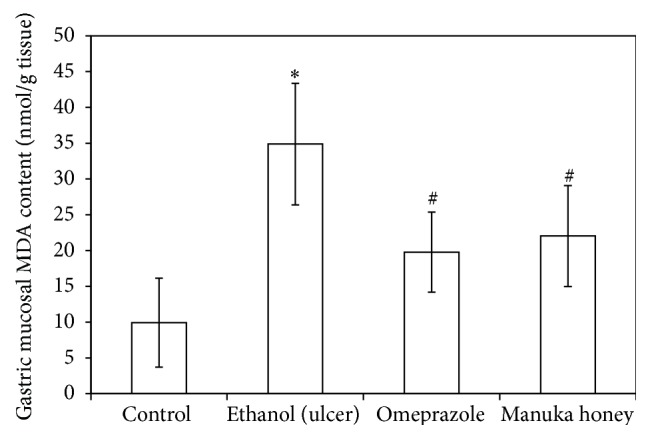
Effect of manuka honey on gastric mucosal lipid peroxide (MDA) content (nmol/g tissue) measured in ethanol-induced gastric ulceration model. Ethanol treated rats were pretreated with either omeprazole (40 mg/kg) or manuka honey (2.5 g/kg). Each value is the mean ± SD (*n* = 6). ^*∗*^Significant versus control group (*P* ≤ 0.05). ^#^Significant versus ethanol (*P* ≤ 0.05).

Treatments of rats with ethanol caused a significant decrease in both gastric mucosal NO and GSH contents (50% and 41%, resp.) as compared to the control contents (*P* = 0.000 and 0.005, resp.) (Figures 5 and 6). On the other hand, treatment of rats with ethanol caused a significant increase (3.5-fold) in gastric mucosal MDA concentration as compared to the controls (*P* = 0.000) (Figure 7). Pretreatment of ulcer-induced rats with both omeprazole and manuka honey significantly increased gastric mucosal NO (109%  and 117%, resp.) (*P* = 0.005 and 0.002) and GSH contents (50% and 90%, resp.) (*P* = 0.000 and 0.004, resp.) (Figures 5 and 6). On the other hand, pretreatment of ulcer-induced rats with both omeprazole and manuka honey significantly decreased gastric mucosal MDA concentrations (43%  and 37%, resp.) as compared to ethanol-injected rats (*P* = 0.005 and 0.017) (Figure 7).

### 3.8. Effect of Manuka Honey on Gastric Mucosal Glutathione Peroxidase (GPx), Superoxide Dismutase (SOD), and Catalase (CAT) Activities

The results of the enzymatic antioxidant analyses are shown in Table 3. Briefly, the activities of CAT were not affected in the different treatment regimens. Treatment of rats with ethanol caused a significant decrease in both gastric mucosal GPx (76%) and SOD activities (31%) as compared to the control rats (*P* = 0.000 and 0.015). Pretreatment of ethanol-injected rats with both omeprazole and manuka honey significantly increased gastric mucosal GPx (~7- and 8-fold, resp.) (*P* = 0.045 and 0.003) and SOD enzyme activity (64%  and 67%, resp.) (*P* = 0.010 and 0.012, resp.).

### 3.9. Effect of Manuka Honey on Plasma Tumour Necrosis Factor Alpha (TNF-*α*), Interleukin-1 Beta (IL-1*β*), and Interleukin-6 (IL-6) Concentrations

The results of the inflammatory cytokine analysis are shown in Table 4. Treatment of rats with ethanol caused a significant increase in plasma TNF-*α*, IL-1*β*, and IL-6 levels (21%, 98%, and 82%, resp.) (*P* = 0.002, 0.000, and 0.000, resp.) as compared to the control group. Pretreatment of ethanol-injected rats with both omeprazole and manuka honey caused a significant decrease in plasma TNF-*α* (38% and 28%) (*P* = 0.000 and 0.000), IL-1*β* (49% and 50%) (*P* = 0.000 and 0.000), and IL-6 (45% and 47%) (*P* = 0.000 and 0.000) as compared to the ulcer-induced rats.

## 4. Discussion

Our results are similar to previously reported results which found that 7 weeks feeding honey did not alter % BWG, food intake, total cholesterol, LDL-cholesterol, or triglyceride levels compared with rats fed a sugar-free diet [30]. Moreover, short-term feeding honey resulted in no increase in HDL-cholesterol levels, compared either with baseline levels or with other dietary treatments [31, 32]. Also, our results are in agreement with a previous study which reported that honey supplementation in nondiabetic rats did not alter the serum concentrations of glucose [33].

The macroscopic and histologic results of this study demonstrated a significant gastroprotective activity of unifloral manuka honey against ethanol-induced stomach ulcer. This is consistent with previous results, which have demonstrated the gastroprotective activity of several types of honey, either unifloral or multifloral, and from different botanical origin [34, 35].

Ethanol-induced stomach ulcer causes an inflammatory response associated with increased neutrophil infiltration and hence results in disturbing the oxidant/antioxidant balance [36]. Ethanol-induced stomach ulcer is usually associated with modulation of the NO pathway [37]. Recently, ethanol has also been found to disturb the inflammatory/anti-inflammatory cytokine balance [8].

Omeprazole was used as a positive control in this study because it is used for treatment of gastric ulcer and has been used in numerous published studies to provide a gastroprotective effect [38–40].

In this study, manuka honey, which is rich in flavonoids, increased the glycoprotein production in the ethanol model of gastric damage. It also preserved the gastric mucosal GSH. Both gastric mucus and GSH serve as protective molecules against gastric mucosal injury [41]. Manuka honey may produce its gastroprotective effect via decreased lipid peroxidation product MDA. This could be because it reserved gastric mucosal GSH contents and increased the formation of gastric mucosal NO. Natural honey prevented gastric mucosal lesions induced by ethanol through the production of nonprotein sulfhydryls and endogenous NO [31]. Manuka honey also increased the antioxidant activity of GPx and SOD enzymes. The antioxidant activity of manuka honey may be attributed to its antioxidant flavonoid content [14, 15]. Many studies have reported the antiulcerogenic properties of flavonoids [42, 43]. The antioxidant activities of flavonoids involve ROS scavenging, transition metal ion chelation, increase of enzymatic and nonenzymatic antioxidants, and reduction of lipid peroxidation [9]. In addition, a recent study revealed that manuka honey was the most effective antioxidant and antibacterial honey compared to both acacia honey and wild carrot honey, possibly because of its high phenol content [13]. Manuka honey has been known to exert antimicrobial function based on its abundant methylglyoxal content. Methylglyoxal on its own is a cytotoxic substance. It would seem, however, that the combination and ratio of methylglyoxal with other components in the manuka honey counteracts the methylglyoxal component from exhibiting such toxicity, because no cytotoxicity is seen in the required testing for FDA registration of manuka honey wound-care products [44].

The GPx activity in the gastric mucosa may be compromised due to ethanol activity, potentially causing an accumulation of hydrogen peroxide levels followed by lipid peroxidation. Thus, the protection of GPx activity in ethanol-treated animals by manuka honey may be due to the preservation of GSH activity and keeping it in a reduced form, in addition to its ability to eliminate hydrogen peroxide and lipid hydroperoxides from the gastric mucosal cell [45]. It has been suggested that GSH plays a role in NO synthesis, either as a reducing cofactor for NO production [46, 47] or more likely by preventing the early inactivation of NO synthesis by ROS or NO itself [48]. NO plays an important role in the control of gastric blood flow as well as in the maintenance of gastric mucosal integrity [18]. NO decreases leukocyte adherence and stimulates gastric mucus secretions [49, 50]. Also, data obtained from both* in vitro* and* in vivo *studies suggested that NO exerts an antiapoptotic effect on rat gastrointestinal mucosal cells [51].

Furthermore, the manuka gastroprotective effect may be due to inhibition of TNF-*α*, IL-1*β*, and IL-6. These proinflammatory cytokines were reported to play a very important role in ethanol-induced gastric ulcer formation, as TNF-*α* is an important modulator of gastric mucosal apoptotic cell death [8].

In conclusion, manuka honey probably prevents ethanol-induced stomach ulcer by protecting the enzymatic (GPx and SOD) and nonenzymatic (GSH and NO) antioxidants, inhibiting lipid peroxidation (MDA), saving mucous glycoprotein, and reducing inflammatory cytokine (TNF-*α*, IL-1*β*, and IL-6) formation.

## Figures and Tables

**Table 1:** Effect of manuka honey on % body weight gain (BWG) and food and water consumption.

Treatment regimen	%BWG	Food consumption (gm/rat/day)	Amount of water ingested (mL/day)
Control	2.55 ± 1.44	29.27 ± 3.72	37.78 ± 7.72
Manuka honey (2.5 gm/kg)	2.78 ± 1.28	26.09 ± 2.94	27.08 ± 9.5

Data are mean ± SD (*n* = 6).

**Table 2:** Effect of manuka honey on serum glucose, triglycerides (TG), total cholesterol, HDL-cholesterol, and LDL-cholesterol.

Treatment regimen	Glucose (mmol/L)	Triglycerides (TG) (mmol/L)	Total cholesterol (mmol/L)	HDL-cholesterol (mmol/L)	LDL-cholesterol (mmol/L)
Control	3.52 ± 1.13	0.37 ± 0.22	1.33 ± 0.41	1.37 ± 0.30	0.30 ± 0.08
Manuka honey (2.5 gm/kg)	3.68 ± 0.46	0.33 ± 0.09	1.19 ± 0.20	1.25 ± 0.13	0.26 ± 0.05

Data are mean ± SD (*n* = 6).

**Table 3:** Effect of manuka honey on gastric mucosal GPx, SOD, and CAT enzyme activity measured in ethanol-induced gastric ulceration model.

Treatment regimen	GPx (U/g tissue)	SOD (U/mg tissue)	CAT (U/g tissue)
Control	629 ± 169	0.48 ± 0.10	0.27 ± 0.06
Ethanol (ulcer)	149 ± 56^a^	0.33 ± 0.069^a^	0.15 ± 0.05^a^
Omeprazole	1028 ± 391^b^	0.54 ± 0.14^b^	0.13 ± 0.04
Manuka honey	1154 ± 283^b^	0.55 ± 0.16^b^	0.18 ± 0.09

Data are mean ± SD (*n* = 6).

^a^Significant versus control (*P* ≤ 0.05).

^b^Significant versus omeprazole (*P* ≤ 0.05).

**Table 4:** Effect of manuka honey on TNF-*α*, IL-1*β*, and IL-6 measured in ethanol-induced gastric ulceration model.

Treatment regimen	TNF-*α* (pg/mL)	IL-1*β* (pg/mL)	IL-6 (pg/mL)
Control	331 ± 35	43 ± 1.5	128 ± 5
Ethanol (ulcer)	421 ± 41^a^	85 ± 0.8^a^	233 ± 10^a^
Omeprazole	304 ± 12^b^	43 ± 2.3^b^	128 ± 5^b^
Manuka honey	306 ± 15^b^	42 ± 1.5^b^	124 ± 5^b^

Data are mean ± SD (*n* = 6).

^a^Significant versus control (*P* ≤ 0.05).

^b^Significant versus omeprazole (*P* ≤ 0.05).
